# Influence of the FIV Status and Chronic Gingivitis on Feline Oral Microbiota

**DOI:** 10.3390/pathogens9050383

**Published:** 2020-05-16

**Authors:** Caitlin E. Older, Márcia de Oliveira Sampaio Gomes, Aline Rodrigues Hoffmann, Mariel Dalmédico Policano, Camila Aparecida Cruz dos Reis, Adriano Bonfim Carregaro, Carlos Eduardo Ambrósio, Valéria Maria Lara Carregaro

**Affiliations:** 1 Department of Veterinary Pathobiology, College of Veterinary Medicine & Biomedical Sciences, Texas A&M University, College Station, TX 77843, USA; colder@cvm.tamu.edu (C.E.O.); arodrigues@cvm.tamu.edu (A.R.H.); 2 Internal Medicine Department, College of Veterinary Medicine and Animal Science, University of São Paulo, São Paulo 05508-270, Brazil; marciadeosg@usp.br; 3 Department of Veterinary Medicine, Faculty of Animal Science and Food Engineering, University of São Paulo, Pirassununga 13635-900, Brazil; mariel.policano@usp.br (M.D.P.); camilareis@usp.br (C.A.C.d.R.); carregaro@usp.br (A.B.C.); ceambrosio@usp.br (C.E.A.)

**Keywords:** microbiota, feline, gingivostomatitis, oral disease, gingivitis, FIV

## Abstract

Feline chronic gingivostomatitis (FCGS) has an unclear pathogenesis with the oral microbiome and viral infections, such as feline immunodeficiency virus (FIV), thought to contribute. Although the relationship between the FIV status and FCGS is not clear, one theory is FIV-induced immune dysregulation could contribute to oral dysbiosis, promoting FCGS development. To further understand the relationship between FCGS, FIV infection, and the oral microbiome, oral cavities of forty cats fitting within 4 groups (FIV- without gingivitis, FIV+ without gingivitis, FIV- with gingivitis, FIV+ with gingivitis) were swabbed. Next generation sequencing targeting the V4 region of the 16s rRNA gene was performed for bacterial community profiling. No differences in diversity were observed, however, analysis of the data in terms of gingivitis revealed differences in the relative abundance of taxa and predicted functional output. *Odoribacter* spp., a bacteria associated with oral disease, was found in higher relative abundances in cats with the highest gingivitis grade. Cats with gingivitis were also found to harbor communities more involved in production of short-chain fatty acids, which have been connected with oral disease. Significant findings associated with the FIV status were few and of low impact, suggesting any connection between the FIV status and FCGS is likely not related to the oral microbiota.

## 1. Introduction

Feline chronic gingivostomatitis (FCGS) has an unclear pathogenesis with many factors thought to contribute, including the oral microbiome. Several bacteria have been found to have different frequencies of isolation in the oral cavity of cats affected by periodontal disease compared to healthy cats, suggesting there may be an important role for the oral microbial communities [1,2,3,4]. While the presence or absence and/or increases in abundances of certain bacteria is not proof of microbiome involvement, some of the same microorganisms that are isolated from the oral cavity of cats affected by oral disease, such as *Porphyromonas* spp. and *Prevotella* spp., are also implicated in human periodontal disease [5]. Although several bacteria have been identified with differential abundance, none have consistently been proposed as important pathogens or commensals that convey benefits to the host. Besides these culture-dependent studies, a single next generation sequencing survey of subgingival samples has also been performed [6]. This study demonstrated several taxa with differential abundance, but unlike the culture-based studies, identified increased diversity in samples from cats affected by FCGS. It seems there is some microbial dysbiosis related to FCGS, but the particular changes that are associated with this disease are not clear.

Viral infections have also long been suspected to contribute to FCGS, primarily due to the increased prevalence of FCGS observed in cats with various viral diseases [7,8,9,10,11,12,13]. Several mechanisms for the involvement of viruses have been proposed. For instance, perhaps, the immune dysregulation associated with viral infection [14] results in an inability to maintain a healthy oral microbiome, allowing pathogens to more easily colonize and infect. The oral microbiome of feline immunodeficiency virus-positive (FIV+) cats has previously been described as dysbiotic, characterized by higher abundances of Fusobacteria, a phylum containing many taxa that are associated with oral disease, as well as increased abundances of Actinobacteria and a different community structure relative to non-infected cats [15]. While our understanding of the oral microbiome in FIV+ cats is limited to this single study, some studies of the oral microbiome in human patients with human immunodeficiency virus (HIV) have also identified alterations in the microbiome [16,17,18]. However, even in HIV+ patients, the role of the microbiome is still unclear, since other studies have found relatively similar microbiomes in both HIV+ and HIV- patients [19,20].

The oral microbiome represents a potentially important intermediate in the connection between FIV infection and FCGS. While studies have provided evidence in support of the role of FIV status and FCGS in modulating the oral microbiome separately, the relationship between FCGS, FIV, and the oral microbiome is still unclear. Furthermore, most studies of the feline oral microbiota have been culture-based. Although culture-dependent studies have greatly added to our understanding, Next-generation sequencing (NGS) can complement these studies by providing a broader representation of the microbial community through circumventing the fastidious nature of many bacteria. Understanding how microbial communities may be altered in disease states can allow for more specific therapeutic development and may be helpful in diagnosing disease and preventing disease flares. Therefore, the aim of this study was to describe the microbial communities in the oral cavity of cats with and without FIV and FCGS using next generation sequencing.

## 2. Results

After quality filtering and removal of sequences classified as Eukarya, Archaea, mitochondria, and Cyanobacteria, a total of 4,165,485 sequences remained for analysis. For diversity analysis, samples were rarefied to 76,829 sequences per sample. No significant differences between the four groups were demonstrated for any of the signalment data (age, sex, city, and diet variables).

**Figure 1:**
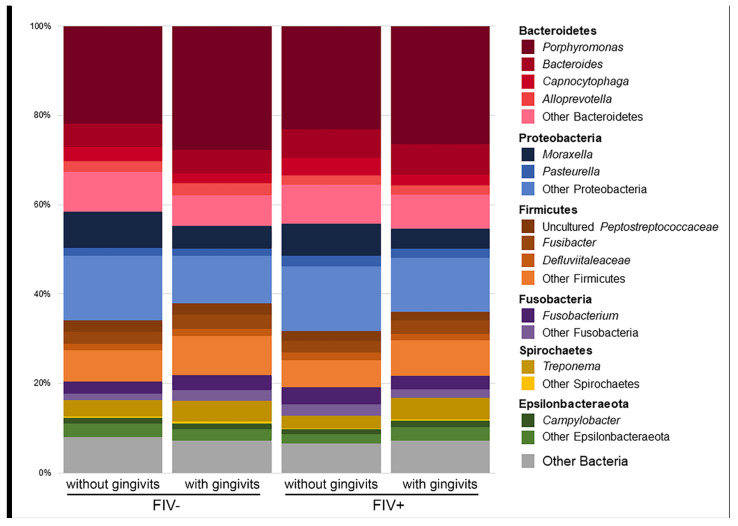
Average relative abundance of the 12 most common genera based on the number of samples where their abundance was > 1%. Bacteroidetes was the most abundant phylum, followed by Firmicutes and Fusobacteria.

Oral bacterial communities were found to be consistent across the four sample groups (FIV- without gingivitis, FIV+ without gingivitis, FIV+ with gingivitis, and FIV- with gingivitis). Alpha and beta diversity analysis did not reveal any differences between the communities, regardless of the metric used. Taxonomic composition and predicted functional output of the microbiome was also consistent. Average relative abundances for each group are shown in Figure 1 (the data for individual samples are shown in Supplementary Figure S1). Analysis of the relative abundance of taxa only identified phylum and class Actinobacteria as having a significant differential abundance (*p* = 0.0162 for phylum and *p* = 0.011 for class). Specifically, FIV+ cats with gingivitis harbored lower relative abundances of this bacteria (average relative abundance of class Actinobacteria = 1.00%) compared to FIV+ cats without gingivitis (*p* = 0.0174 and *p* = 0.0108, average relative abundance = 1.71%) and FIV- cats without gingivitis (*p* = 0.0078 and *p* = 0.006, average relative abundance = 2.28%).

Besides comparing these four groups, the data were also analyzed in terms of the FIV status, gingivitis status, gingivitis grade, and diet. Analyzing these variables also did not result in any significant differences between groups with respect to alpha or beta diversity, suggesting the diversity of the oral microbiota is stable in this group of cats. However, analysis of the relative abundance of taxa and predicted functional output of the microbiome revealed several differences in the oral microbial communities, which are described below.

### 2.1. Effect of the FIV Status

Corynebacteriaceae and Corynebacteriales were found in higher relative abundances in FIV- cats relative to FIV+ cats (Linear discriminant analysis (LDA) score = 2.68, *p* = 0.0074 for both). Analysis of predicted functions of all samples (n = 40) did not result in differences between cats of different FIV status. However, when only cats with gingivitis (n = 20) were analyzed and compared based on the FIV status, FIV- cats had communities that were more prone to participate in pyruvate fermentation to acetone (LDA score = 2.55, *p* = 0.0015).

### 2.2. Effect of the Gingivitis Status

**Figure 2:**
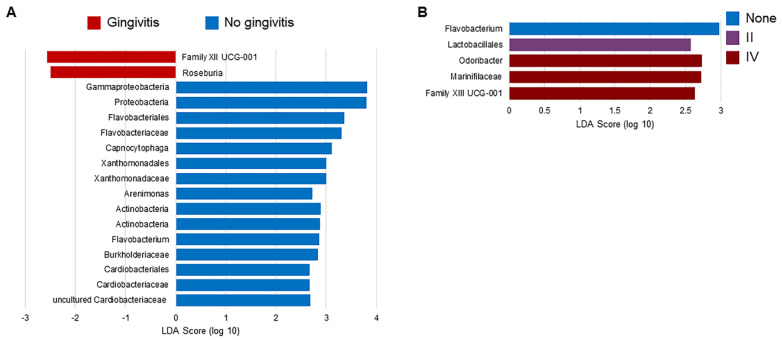
Linear discriminant analysis Effect Size (LEfSe) results analyzing the relative abundance of taxa with respect to (**A**) the gingivitis status and (**B**) the gingivitis grade among all samples. The taxa shown were found to have higher relative abundances in the labelled group relative to others with *p* < 0.01.

**Figure 3:**
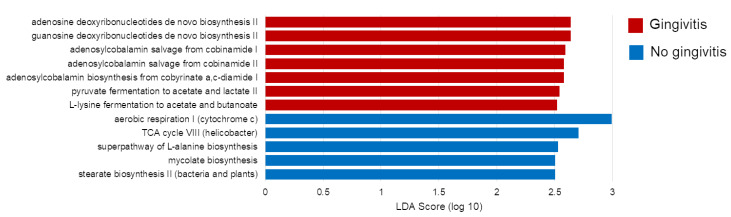
Linear discriminant analysis Effect Size (LEfSe) results analyzing differences in predicted microbial functions between cats with and without gingivitis.

Regardless of the FIV status, *Flavobacterium* spp. were found in an increased relative abundance in cats without gingivitis relative to cats with gingivitis (LDA Score = 2.86, *p* < 0.001, Supplementary Figure S2). Several other bacteria were found to have an increased relative abundance in cats without gingivitis, including *Capnocytophaga* spp. (LDA Score = 3.11, *p* = 0.0041)*,* another genus within the Flavobacteriaceae family (Figure 2A). When samples were evaluated in terms of the gingivitis grade, cats with a gingivitis grade of IV had higher relative abundances of *Odoribacter* spp. (LDA Score = 2.73, *p* < 0.001, Figure 2B). Differences in composition associated with gingivitis also translated to differences in predicted functions of the microbiome. Cats with gingivitis had more bacteria associated with purine and vitamin B12 biosynthesis and fermentation to short-chain fatty acids, while cats without gingivitis had more bacteria associated with energy production and biosynthesis of amino acids and fatty acids (Figure 3). Cats with a gingivitis grade of II had increased palmitate biosynthesis II (LDA Score = 2.94, *p* = 0.0064). 

### 2.3. Effect of Diet

For the diet analyses, only cats without gingivitis were included, since gingivitis was found to be impactful on oral communities. Inclusion of the functional ingredients did not appear to result in microbiome differences, however, diet regimen and specific feed did have some influence. Cats fed diet regimen A (dry food) only had higher abundances of UCG-011, a genus within family Defluviitaleaceae (LDA Score = 2.92, *p* = 0.0034). Cats fed feed 2, which had the lowest lipid content of all the diets and was one of the diets that contained two prebiotics, had increased saturated fatty acid elongation (LDA Score = 2.80, *p* = 0.0091). Cats fed feed 3, which did not contain prebiotics, had the lowest amount of proteins, and had one of the lowest amounts of lipids, harbored communities predicted to be involved in increased lipid IVA biosynthesis (LDA Score = 2.57, *p* = 0.0089).

### 2.4. Other Factors

The effect of age and sex was also evaluated, with only the LEfSe analysis of predicted microbial functions revealing some significant findings. Cats fitting into the senior age group (ages 13–15 years) had higher relative abundances of bacteria involved in L-methionine and L-arginine biosynthesis (LDA Score > 2.66, *p* < 0.01), while mature cats (ages 7–9 years) had higher relative abundances of bacteria involved in glucose and xylose degradation (LDA Score = 2.70, *p* = 0.0091). Female cats were also found to have higher relative abundances of bacteria involved in L-arginine synthesis (LDA Score = 2.53, *p* = 0.0043).

## 3. Discussion

In this study, we found the feline oral microbiota to be stable in terms of diversity, regardless of the FIV status, gingivitis status, diet, and even intrinsic factors, such as age and sex. While alpha and beta diversity were unaffected, composition and predicted functional output of the microbiota were more sensitive to these factors. In particular, gingivitis seemed to be influential on community composition and community functional output.

We were especially interested in the influence of the FIV status on the oral microbiome due to a suspected relationship between FIV and gingivitis. While FIV cats seem to have higher incidences of FCGS [7,8,9,10,11,12], it is unclear why this may occur. One theory is that the deficient immune system could result in physiological changes within the oral cavity that would support an altered microbiome. In humans, studies have found evidence of oral bacteria and their products [21] affecting activation and replication of Epstein–Barr virus and HIV [22], demonstrating that not only what bacteria are present, but also what they produce and how they interact with viruses may be important.

Within our study, the FIV status had a minimal effect on the oral microbiome. Only two taxa, Corynebacteriaceae and Corynebacteriales, had a higher relative abundance in FIV- cats without gingivitis. Given the low average relative abundance of these bacteria (less than 1% in both groups), the biological impact of this finding is questionable. A previous study [15] also evaluated the oral microbiomes in FIV+ and FIV- cats, and found phyla Fusobacteria and Actinobacteria were identified in higher relative abundances in FIV+ cats. In the present study, although these taxa were found in low abundances overall, differences in the relative abundance of Actinobacteria at the phylum and class level were found between the four groups. FIV+ cats with gingivitis had lower relative abundances of Actinobacteria compared to cats without gingivitis, regardless of the FIV status. Although Actinobacteria may be relevant in some human oral diseases [15], it’s very low abundance in both the previously mentioned study and our own (average relative abundance of class Actinobacteria across all samples = 1.63%), as well as the conflicting results, makes the significance of Actinobacteria in the feline oral cavity unknown.

Our results do not suggest a strong influence of the FIV status on the composition of the oral microbiota, but it is possible our study was just not able to uncover evidence of this link. Differences in composition of the microbiota may exist at a level (e.g., species or strain) which is not discernable with the sequencing technology utilized in this study. Additionally, microbial activity is partly dependent on the status of the host [23]; even if there are no differences in composition, there may be differences in functional output of the microbiota which could not be derived from the predictive methods we used. Further research would be needed to resolve the dynamic roles oral cavity-inhabiting microbes may take on in the context of the FIV status.

Gingivitis status had a higher impact on the microbiome, with 17 taxa identified as differentially abundant with LEfSe. Some notable microbes with differential abundance were genera *Flavobacterium* and *Capnocytophaga. Flavobacterium* spp. were found in higher relative abundances in cats without gingivitis, regardless of the FIV status. *Capnocytophaga* spp. was also present in higher relative abundances of cats without gingivitis. Although *Capnocytophaga* spp. is often associated with disease due to cat bites, it is a normal inhabitant of the feline oral microbiota [24]. The higher relative abundance of these bacteria in the healthy feline oral cavity within the presented data is concordant with two previous studies [3,6].

Besides *Capnocytophaga* spp., other bacteria have been suggested as potential contributors to FCGS. Several novel bacteria have been identified in the oral cavity of cats, which were proposed to be relevant to the disease process [25]. This same research group also suggested that *Pasteurella multocida* subsp. *multocida* may be relevant to FCGS [3]. Bacterial genus *Pasteurella* is recognized as a normal inhabitant of the feline oral cavity, but they can also be a cause of infections associated with cat bites [26]. In their study utilizing sequencing of cultured isolates, they found *P. multocida* subsp. *multocida* was prevalent in the oral cavity of cats affected with FCGS and absent in normal cats. Several culture-based studies [1,4,27,28], a culture-independent survey of the subgingival microbiota [6], and the present study, however, did not identify increased relative abundances or frequency of isolation from affected cats. Considering these results and the small sample size included in the initial culture-based study, there does not seem to be enough evidence to implicate *Pasteurella* spp. as an important pathogen in FCGS.

Gingivitis also had an influence on predicted functional output of the microbiota. Twelve microbial pathways were found to be predicted at different levels based on the gingivitis status. Cats with gingivitis had more bacteria known to be involved in two pathways, fermentation to acetate and butanoate. Bacteria-derived short-chain fatty acids (SCFAs), such as acetate and butanoate, are typically found in higher amounts in the oral cavity of patients with periodontal disease [29,30,31]. In addition to being associated with inflammation in these patients, research discovered these molecules have the ability to negatively affect host cell proliferation and activity [32]. To our knowledge, this same association and mechanism of SCFAs in feline oral disease have not been evaluated. Our results suggest SCFAs may be important in the pathogenesis of FCGS and are worth further researching for the potential of using specific SCFAs as biomarkers.

Evaluating cats based on the gingivitis grade also revealed multiple taxa as differentially abundant. Of interest to us was *Odoribacter* spp., which was found in significantly higher relative abundances in the oral cavity of cats with grade IV gingivitis relative to all other grades. The recently described genus was created upon isolating a novel bacterial species, *Odoribacter denticanis*, from the oral cavity of a dog with periodontal disease and was named accordingly due to the associated foul odor. In a mouse model of periodontal disease, this bacteria was able to cause oral disease, indicating it may be a relevant pathogen in animals [33]. This species was also found in cats with periodontal disease in a study sequencing plaque-inhabiting bacteria [34]. The higher relative abundance of this microbe in the cats with severe gingivitis in this study and another one [6] is particularly intriguing. If future studies confirm these bacteria are important in the development of oral disease in cats, it could serve as a target, either through diet changes or therapeutics, which could be useful in managing FCGS and potentially the malodor associated with this condition.

In studying the oral microbiome, the diet is often a factor of particular interest. Nutrients could shape oral communities through host or microbial metabolism. Although not the main objective of this study, diet was analyzed with respect to the diet regimen (dry food, wet food, mix of dry and homemade food), the specific feed, and the inclusion of functional ingredients. Diet regimen (wet vs. dry food) had previously been shown to influence the microbial communities in a feline oral cavity, with cats fed dry food having more diverse communities than cats fed wet food and with several differences in composition of the communities [35]. Within the present study, a couple of differences in the composition of these communities and their predicted functional output were observed, but no significant difference in diversity was found. Diet certainly has an influence on the oral microbiome, but, as emphasized previously, further research is needed to determine the impact it may have on feline oral health [35].

Within this study, several differences in composition of the oral communities were observed. Although identifying bacteria with different relative abundances between healthy and affected animals is important in understanding which microbes may be relevant to disease, it is important to recognize that many of these bacteria are not solely pathogenic or beneficial. As previously mentioned, microbial activity depends on the environment, including other microbes they may be cohabiting or competing with and the health status of the host. Further research is needed to clarify what the role of the differentially abundant bacteria in disease may be. Future studies will need to evaluate the microbe–microbe interactions that occur under different physiological conditions and look into what products these microbes may be producing. The microbiota can generate a vast range of metabolites, and their identification and characterization is a challenge in metabolomics. In the presented findings, we utilized PICRUSt2 to predict the functional output of the microbiome based on the taxa that are found. Methods that can directly evaluate the activity or capabilities of microbes, such as microbial transcriptomics or metabolomics, would be incredibly useful in better understanding what pathways may be up- or down-regulated in affected animals. The possible changes in bacterial metabolism affect how the host is able to respond to microbes or the regulation of the immune response, potentially resulting in increased inflammation even in the absence of dysbiosis.

## 4. Materials and Methods

### 4.1. Sample Collection and DNA Extraction

Forty client-owned cats from four different groups (FIV- without gingivitis, FIV- with gingivitis, FIV+ with gingivitis, FIV+ without gingivitis; ten cats per group) were included in this study. Cats were examined by a veterinarian at the University of São Paulo (USP) veterinary hospital in Pirassununga, São Paulo, Brazil. Approval for this study was obtained from the Bioethical Committee of the Faculdade de Zootecnia e Engenharia de Alimentos (FZEA), Universidade de São Paulo, Pirassununga, São Paulo (protocol number 14.1.1500.74.6), and informed consent was obtained from each owner. Gingivostomatitis was scored as previously described [15,36], with some modifications: 0 = normal gingiva, 1 = slight inflammation, 2 = moderate inflammation, 3 = severe inflammation and bleeding, and 4 = severe inflammation, bleeding, and missing teeth. Testing for FIV and FeLV infection were performed in duplicate with the SNAP FIV/FeLV Combo Test (Idexx Laboratories, Wetbrook, ME, USA). All cats were at least 1 year old, spayed or neutered, had not received any antimicrobial or anti-inflammatory drugs within 30 days of sampling, and had negative FeLV results. At the time of sampling, the information regarding approximate age (young = 1–3 years, adult = 4–6 years, mature = 7–9 years, old = 10–12 years, senior = 13–15 years), sex, diet regimen (A = dry food, B = dry and wet food, C = mix of dry and homemade food), specific feed, if the diet included prebiotics (mannan oligosaccharides and inulin), environment (indoor or outdoor), vaccine history, and disease history was recorded and is provided in Supplementary Table S1. Sample groups were not significantly different with respect to signalment data. From each cat, two Isohelix buccal swabs (Cell Projects Ltd., Kent, UK) were swabbed from the gums of both the upper and the lower dental arcades, tongue, palate, and teeth, 10 times on each side of each swab. The two swabs were then stored in a MO BIO PowerBead tube (MO BIO Laboratories, Carlsbad, CA, USA) and extracted using a modified protocol with a MO BIO PowerSoil DNA Isolation Kit (MO BIO Laboratories).

### 4.2. Next Generation Sequencing

PCR reactions targeting the V4 region of the 16s rRNA gene consisted of 10 μL of GoTaq^®^ Colorless Master Mix 2× (Promega, Madison, WI, USA), 0.3 μM forward primer (515F: GTGCCAGCMGCCGCGGTAA), 0.3 μM reverse primer (806R: GGACTACHVGGGTWTC), 20 ng of genomic DNA, and 20 μL of water and were run on a Veriti Thermal Cycler (Applied Biosystems, Foster City, CA). These reactions were run with an initial denaturation at 94 °C for 3 min, 29 cycles of 94 °C for 45 s, 50 °C for 1 min, 72 °C for 1 min and 30 s, followed by a final extension of 10 min at 72 °C. PCR products were then checked on a 2% agarose gel.

For library preparation, PCR triplicates of each sample were pooled in an aliquot and purified using Agencourt AMPure XP magnetic beads (Beckman Coulter, Brea, CA, USA). Libraries were quantified by real-time PCR using a KAPA Library Quantification Kit for Illumina sequencing (Kapa Biosystems, Wilmington, MA, USA). Samples were then normalized to 3 nM prior to sequencing on an Illumina MiSeq sequencing system (Illumina, Inc., San Diego, CA, USA) at BPI Biotecnologia (São Paulo, Brazil). Raw sequences are accessible under BioProject ID PRJNA580001 in the NCBI Sequence Read Archive.

### 4.3. Sequence Processing

Primers were removed from the resulting sequences using cutadapt [37] and were further processed in QIIME 2 (2018.6) [38], where sequence dereplication and chimera removal were performed with VSEARCH [39] and UCHIME [40] and taxonomic assignments were determined using the SILVA database (version 132 release) [41] with a scikit-learn classifier [42]. The resulting data were also processed with PICRUSt2 (QIIME 2 plugin) [43] to generate predicted functional output of the microbiota.

### 4.4. Data Analysis

Data were analyzed in terms of the group (FIV-, FIV+, FIV- with gingivitis, FIV+ with gingivitis), FIV status, gingivitis status, gingivitis grade, age, sex, city, and diet. More specifically, the diet was analyzed in terms of the diet regimen (A = dry food, B = dry and wet food, C = mix of dry and homemade food), specific feed (Supplementary Table S2), and inclusion of prebiotics in diet (mannan oligosaccharides and inulin). Taxonomic and predicted functional data were analyzed with the Linear discriminant analysis (LDA) Effect Size (LEfSe) algorithm [44] and with Wilcoxon tests, or Kruskal–Wallis tests where appropriate, in JMP Pro 14 (SAS Institute, Cary, NC). Alpha diversity was calculated using the Chao1 diversity index, Faith’s phylogenetic diversity, observed OTUs (operational taxonomic units), Pielou’s evenness, and Shannon diversity index, with the resulting data analyzed using Wilcoxon or Kruskal-Wallis tests. Beta diversity was calculated with the Bray–Curtis dissimilarity, Jaccard distance, and weighted and unweighted UniFrac metrics [45]. Resulting distance matrices were analyzed using ANOSIM (Analysis of similarities) tests in R [46] with the vegan package [47]. Where appropriate, the Benjamini–Hochberg *p*-value correction was performed [48].

## 5. Conclusions

Although decreases in diversity are thought to be associated with diseased states, this was not observed within the present study when analyzing the FIV status, gingivitis status, or both. Even when considering other factors, such as age, sex, and diet, diversity of the feline bacterial oral microbiota was consistent. While differences in alpha and beta diversity were not observed, several taxa were identified as differentially abundant with respect to some of the factors analyzed. The FIV status seemed to have only a minor influence on the oral microbiota of the cats sampled in this study, with only a few differences in relative abundance of taxa with unknown biological impact identified. In contrast, gingivitis had a notable impact on the oral communities with some changes that are intelligible considering the clinical features and the bacteria. For example, *Odoribacter* spp. was found in cats with the highest gingivitis score and is associated with oral malodor, a common clinical sign in cats with gingivitis. Additionally, gingivitis was influential on predicted output of the microbiota, with populations more associated with production of short-chain fatty acids, which have previously been associated with oral disease severity, in cats with gingivitis. Our results indicate there is no clear evidence to indicate gingivitis and the FIV status have some synergistic impact on the microbiota, but gingivitis certainly has some influence. Further research into gingivitis-related dysbiosis may unveil useful targets for therapeutic intervention and prevention.

## Supplementary Materials

The following are available online at https://www.mdpi.com/2076-0817/9/5/383/s1: Figure S1. Relative abundance of taxa in individual samples; Figure S2. Linear discriminant analysis Effect Size (LEfSe) results analyzing the relative abundance of taxa with respect to the gingivitis status in A) FIV+ cats and B) FIV- cats; Table S1. Signalment and history of study participants; and Table S2. Diet information.

## References

[1] Mallonee D.H., Harvey C.E., Venner M., Hammond B.F. (1988). Bacteriology of periodontal disease in the cat. Arch. Oral Biol..

[2] Love D.N., Johnson J.L., Moore L.V. (1989). Bacteroides species from the oral cavity and oral-associated diseases of cats. Vet. Microbiol..

[3] Dolieslager S.M., Riggio M.P., Lennon A., Lappin D.F., Johnston N., Taylor D., Bennett D. (2011). Identification of bacteria associated with feline chronic gingivostomatitis using culture-dependent and culture-independent methods. Vet. Microbiol..

[4] Nakanishi H., Furuya M., Soma T., Hayashiuchi Y., Yoshiuchi R., Matsubayashi M., Tani H., Sasai K. (2019). Prevalence of microorganisms associated with feline gingivostomatitis. J. Feline Med. Surg..

[5] Costalonga M., Herzberg M.C. (2014). The oral microbiome and the immunobiology of periodontal disease and caries. Immunol. Lett..

[6] Rodrigues M.X., Bicalho R.C., Fiani N., Lima S.F., Peralta S. (2019). The subgingival microbial community of feline periodontitis and gingivostomatitis: Characterization and comparison between diseased and healthy cats. Sci. Rep..

[7] Tenorio A.P., Franti C.E., Madewell B.R., Pedersen N.C. (1991). Chronic oral infections of cats and their relationship to persistent oral carriage of feline calici-, immunodeficiency, or leukemia viruses. Vet. Immunol. Immunopathol..

[8] Knowles J.O., Gaskell R.M., Gaskell C.J., Harvey C.E., Lutz H. (1989). Prevalence of feline calicivirus, feline leukaemia virus and antibodies to FIV in cats with chronic stomatitis. Vet. Rec..

[9] Quimby J.M., Elston T., Hawley J., Brewer M., Miller A., Lappin M.R. (2008). Evaluation of the association of Bartonella species, feline herpesvirus 1, feline calicivirus, feline leukemia virus and feline immunodeficiency virus with chronic feline gingivostomatitis. J. Feline Med. Surg..

[10] Belgard S., Truyen U., Thibault J.C., Sauter-Louis C., Hartmann K. (2010). Relevance of feline calicivirus, feline immunodeficiency virus, feline leukemia virus, feline herpesvirus and Bartonella henselae in cats with chronic gingivostomatitis. Berl. Munch Tierarztl. Wochenschr..

[11] Dowers K.L., Hawley J.R., Brewer M.M., Morris A.K., Radecki S.V., Lappin M.R. (2010). Association of Bartonella species, feline calicivirus, and feline herpesvirus 1 infection with gingivostomatitis in cats. J. Feline Med. Surg..

[12] Lommer M.J. (2013). Oral inflammation in small animals. Vet. Clin. N. Am. Small Anim. Pract..

[13] Kornya M.R., Little S.E., Scherk M.A., Sears W.C., Bienzle D. (2014). Association between oral health status and retrovirus test results in cats. J. Am. Vet. Med. Assoc..

[14] Taniwaki S.A., Figueiredo A.S., Araujo J.P. (2013). Virus-host interaction in feline immunodeficiency virus (FIV) infection. Comp. Immunol. Microbiol. Infect. Dis..

[15] Weese S.J., Nichols J., Jalali M., Litster A. (2015). The oral and conjunctival microbiotas in cats with and without feline immunodeficiency virus infection. Vet. Res..

[16] Goncalves L.S., Soares Ferreira S.M., Souza C.O., Souto R., Colombo A.P. (2007). Clinical and microbiological profiles of human immunodeficiency virus (HIV)-seropositive Brazilians undergoing highly active antiretroviral therapy and HIV-seronegative Brazilians with chronic periodontitis. J. Periodontol..

[17] Mukherjee P.K., Chandra J., Retuerto M., Sikaroodi M., Brown R.E., Jurevic R., Salata R.A., Lederman M.M., Gillevet P.M., Ghannoum M.A. (2014). Oral mycobiome analysis of HIV-infected patients: Identification of Pichia as an antagonist of opportunistic fungi. PLoS Pathog..

[18] Li Y., Saxena D., Chen Z., Liu G., Abrams W.R., Phelan J.A., Norman R.G., Fisch G.S., Corby P.M., Dewhirst F. (2014). HIV infection and microbial diversity in saliva. J. Clin. Microbiol..

[19] Kistler J.O., Arirachakaran P., Poovorawan Y., Dahlen G., Wade W.G. (2015). The oral microbiome in human immunodeficiency virus (HIV)-positive individuals. J. Med. Microbiol..

[20] Beck J.M., Schloss P.D., Venkataraman A., Twigg H., Jablonski K.A., Bushman F.D., Campbell T.B., Charlson E.S., Collman R.G., Crothers K. (2015). Multicenter Comparison of Lung and Oral Microbiomes of HIV-infected and HIV-uninfected Individuals. Am. J. Respir. Crit. Care Med..

[21] Frisan T., Nagy N., Chioureas D., Terol M., Grasso F., Masucci M.G. (2019). A bacterial genotoxin causes virus reactivation and genomic instability in Epstein-Barr virus infected epithelial cells pointing to a role of co-infection in viral oncogenesis. Int. J. Cancer.

[22] Gonzalez O.A., Li M., Ebersole J.L., Huang C.B. (2010). HIV-1 reactivation induced by the periodontal pathogens Fusobacterium nucleatum and Porphyromonas gingivalis involves Toll-like receptor 2 [corrected] and 9 activation in monocytes/macrophages. Clin. Vaccine Immunol..

[23] Hajishengallis G., Lamont R.J. (2012). Beyond the red complex and into more complexity: The polymicrobial synergy and dysbiosis (PSD) model of periodontal disease etiology. Mol. Oral Microbiol..

[24] Lloret A., Egberink H., Addie D., Belák S., Boucraut-Baralon C., Frymus T., Pennisi M.G., Gruffydd-Jones T., Hartmann K., Hosie M.J. (2013). Capnocytophaga Canimorsus Infection in Cats: ABCD guidelines on prevention and management. J. Feline Med. Surg..

[25] Dolieslager S.M., Bennett D., Johnston N., Riggio M.P. (2013). Novel bacterial phylotypes associated with the healthy feline oral cavity and feline chronic gingivostomatitis. Res. Vet. Sci..

[26] Lloret A., Egberink H., Addie D., Belak S., Boucraut-Baralon C., Frymus T., Gruffydd-Jones T., Hartmann K., Hosie M.J., Lutz H. (2013). Pasteurella multocida infection in cats: ABCD guidelines on prevention and management. J. Feline Med. Surg..

[27] Love D.N., Vekselstein R., Collings S. (1990). The obligate and facultatively anaerobic bacterial flora of the normal feline gingival margin. Vet. Microbiol..

[28] Whyte A., Gracia A., Bonastre C., Tejedor M.T., Whyte J., Monteagudo L.V., Simon C. (2017). Oral Disease and Microbiota in Free-Roaming Cats. Top. Companion Anim Med..

[29] Qiqiang L., Huanxin M., Xuejun G. (2012). Longitudinal study of volatile fatty acids in the gingival crevicular fluid of patients with periodontitis before and after nonsurgical therapy. J. Periodontal. Res..

[30] Rzeznik M., Triba M.N., Levy P., Jungo S., Botosoa E., Duchemann B., Le Moyec L., Bernaudin J.F., Savarin P., Guez D. (2017). Identification of a discriminative metabolomic fingerprint of potential clinical relevance in saliva of patients with periodontitis using 1H nuclear magnetic resonance (NMR) spectroscopy. PLoS ONE.

[31] Niederman R., Buyle-Bodin Y., Lu B.Y., Robinson P., Naleway C. (1997). Short-chain carboxylic acid concentration in human gingival crevicular fluid. J. Dent. Res..

[32] Jeng J.H., Chan C.P., Ho Y.S., Lan W.H., Hsieh C.C., Chang M.C. (1999). Effects of butyrate and propionate on the adhesion, growth, cell cycle kinetics, and protein synthesis of cultured human gingival fibroblasts. J. Periodontol..

[33] Hardham J.M., King K.W., Dreier K., Wong J., Strietzel C., Eversole R.R., Sfintescu C., Evans R.T. (2008). Transfer of Bacteroides splanchnicus to Odoribacter gen. nov. as Odoribacter splanchnicus comb. nov., and description of Odoribacter denticanis sp. nov., isolated from the crevicular spaces of canine periodontitis patients. Int. J. Syst. Evol. Microbiol..

[34] Perez-Salcedo L., Laguna E., Sanchez M.C., Marin M.J., O’Connor A., Gonzalez I., Sanz M., Herrera D. (2015). Molecular identification of black-pigmented bacteria from subgingival samples of cats suffering from periodontal disease. J. Small Anim. Pract..

[35] Adler C.J., Malik R., Browne G.V., Norris J.M. (2016). Diet may influence the oral microbiome composition in cats. Microbiome.

[36] Harley R., Gruffydd-Jones T.J., Day M.J. (2003). Salivary and serum immunoglobulin levels in cats with chronic gingivostomatitis. Vet. Rec..

[37] Martin M. (2011). Cutadapt removes adapter sequences from high-throughput sequencing reads. EMBnet. J..

[38] Bolyen E., Rideout J.R., Dillon M.R., Bokulich N.A., Abnet C.C., Al-Ghalith G.A., Alexander H., Alm E.J., Arumugam M., Asnicar F. (2019). Reproducible, interactive, scalable and extensible microbiome data science using QIIME 2. Nat. Biotechnol..

[39] Rognes T., Flouri T., Nichols B., Quince C., Mahe F. (2016). VSEARCH: A versatile open source tool for metagenomics. PeerJ.

[40] Edgar R.C., Haas B.J., Clemente J.C., Quince C., Knight R. (2011). UCHIME improves sensitivity and speed of chimera detection. Bioinformatics.

[41] Quast C., Pruesse E., Yilmaz P., Gerken J., Schweer T., Yarza P., Peplies J., Glockner F.O. (2013). The SILVA ribosomal RNA gene database project: Improved data processing and web-based tools. Nucleic Acids Res..

[42] Abraham A., Pedregosa F., Eickenberg M., Gervais P., Mueller A., Kossaifi J., Gramfort A., Thirion B., Varoquaux G. (2014). Machine learning for neuroimaging with scikit-learn. Front. Neuroinform..

[43] Douglas G.M., Maffei V.J., Zaneveld J., Yurgel S.N., Brown J.R., Taylor C.M., Huttenhower C., Langille M.G.I. (2019). PICRUSt2: An improved and extensible approach for metagenome inference. bioRxiv.

[44] Segata N., Izard J., Waldron L., Gevers D., Miropolsky L., Garrett W.S., Huttenhower C. (2011). Metagenomic biomarker discovery and explanation. Genome Biol..

[45] Lozupone C., Knight R. (2005). UniFrac: A new phylogenetic method for comparing microbial communities. Appl. Environ. Microbiol..

[46] Team R.C. (2019). R: A Language and Environment for Statistical Computing.

[47] Oksanen J., Blanchet F.G., Friendly M., Kindt R., Legendre P., McGlinn D., Minchin P.R., O’Hara R.B., Simpson G.L., Solymos P. (2019). Vegan: Community Ecology Package. R Package Version 2.5-5. https://cran.r-project.org/web/packages/vegan/vegan.pdf.

[48] Benjamini Y., Hochberg Y. (1995). Controlling the false discovery rate: A practical and powerful approach to multiple testing. J. R. Stat. Soc. Ser. B (Stat. Methodol.).

