# Supplementary figures and images for: Influence of the FIV Status and Chronic Gingivitis on Feline Oral Microbiota
Source: Pathogens. 2020 May 16;9(5):383. doi: 10.3390/pathogens9050383 (PMC7281021; doi:10.3390/pathogens9050383)

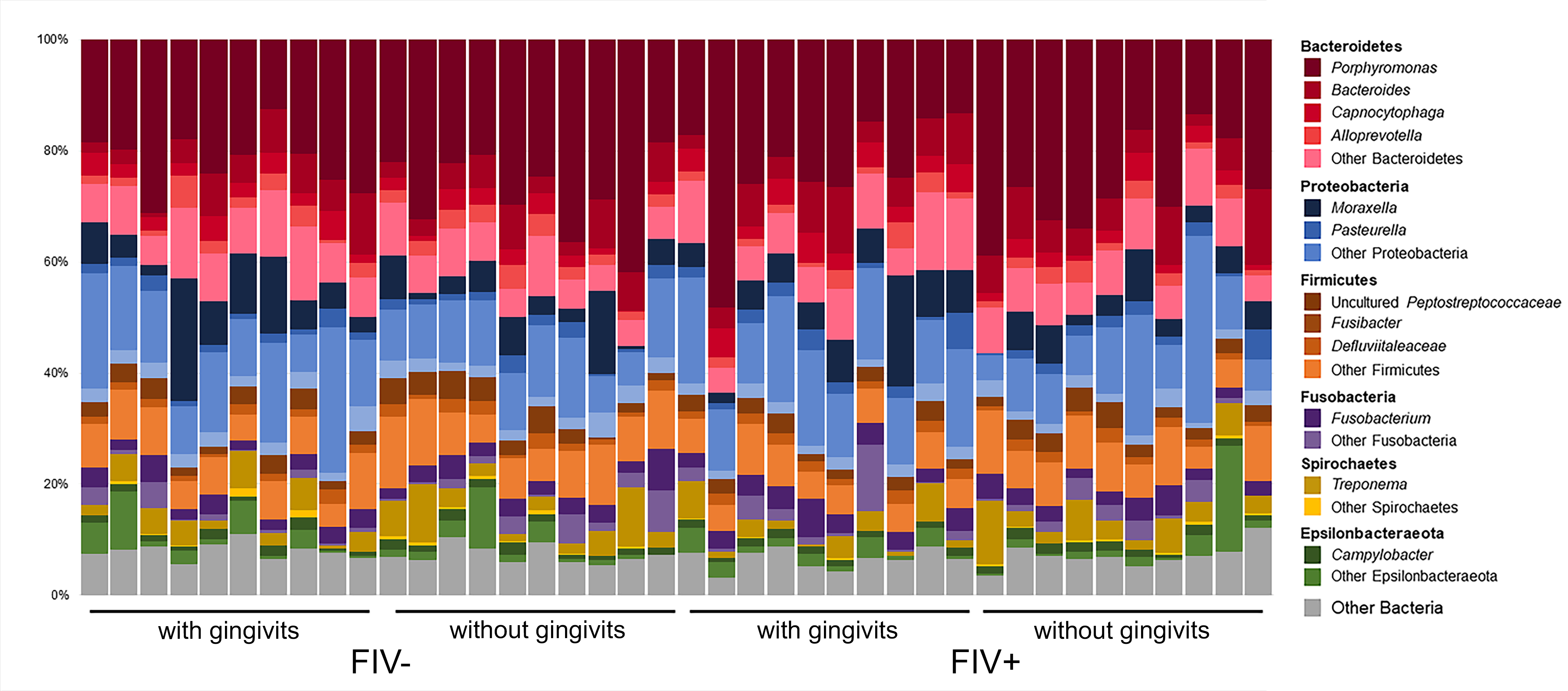

Supplement: Supplementary file 1 [file pathogens-09-00383-s001.zip › supplement CEO et al/Fig S1_revised.tif]

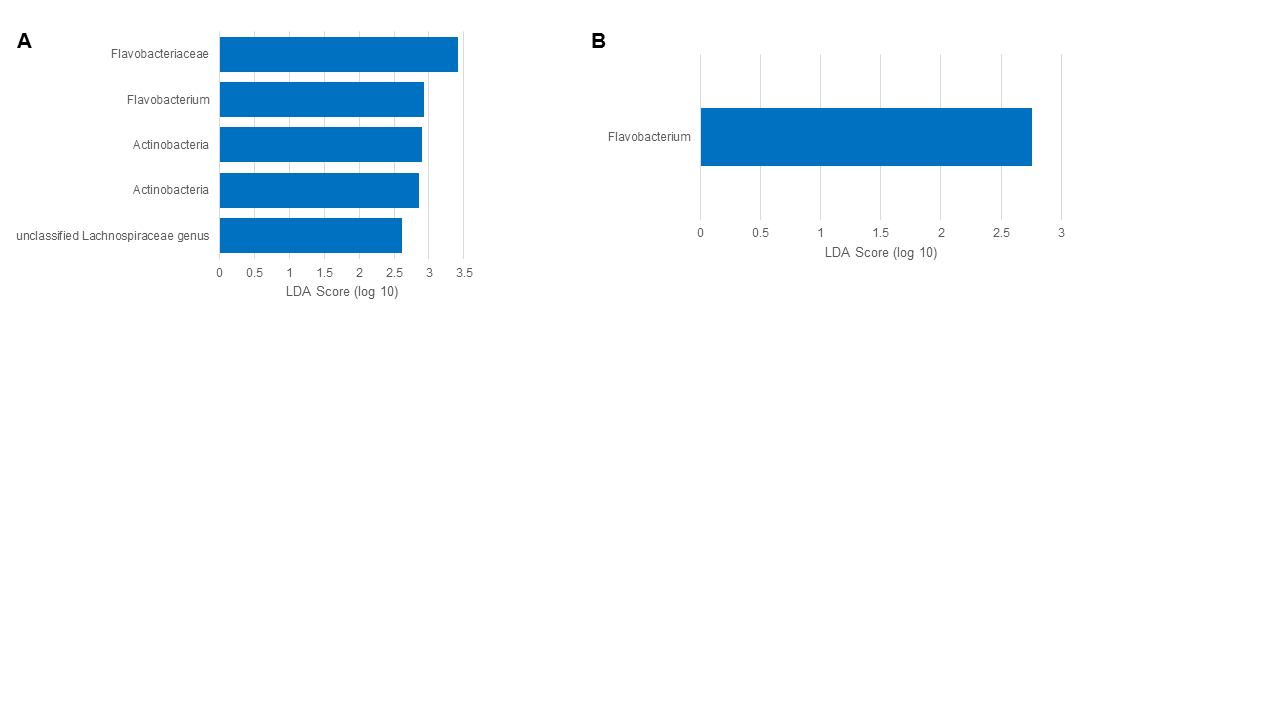

Supplement: Supplementary file 1 [file pathogens-09-00383-s001.zip › supplement CEO et al/Fig S2.TIF]
